# The effectiveness of integrated treatment in patients with substance use disorders co-occurring with anxiety and/or depression - a group randomized trial

**DOI:** 10.1186/1471-244X-14-67

**Published:** 2014-03-05

**Authors:** Linda E Wüsthoff, Helge Waal, Rolf W Gråwe

**Affiliations:** 1 Norwegian Centre for Addiction Research, Institute of Clinical Medicine, University of Oslo, Oslo, Norway; 2 The Agency of Welfare and Social Services, the City of Oslo, Oslo, Norway; 3 Department of Research and Development, Clinic for Substance Use and Addiction Medicine, St. Olav University Hospital, Trondheim, Norway

**Keywords:** Co-occurring disorders, Mental health disorder, Anxiety, Depression, Substance use disorder, Randomized controlled trial, Group-randomized, Outpatient, Community mental health centre, Integrated treatment

## Abstract

**Background:**

Integrated Treatment (IT) has proved effective in treating patients with Substance Use Disorders (SUD) co-occurring with severe Mental Disorders (MD), less is known about the effectiveness of IT for patients with SUD co-occurring with less severe MD.

The aim of this study was to investigate the effectiveness of IT for patients with SUD co-occurring with anxiety and/or depression on the following parameters:

1. The use of substances, as measured by the Alcohol Use Identification Test (AUDIT), the Drug Use Identification Test (DUDIT), and the Addiction Severity Index (EuropASI).

2. The severity of psychiatric symptoms, as measured by the Symptom Check List 90 r (SCL 90R).

3. The client’s motivation for changing his/her substance use behaviour, as measured by the Substance Abuse Treatment Scale (SATSr).

**Methods:**

This is a group randomized clinical trial comparing the effectiveness of IT to treatment as usual in Community Mental Health Centres (CMHCs). Five CMHCs were drawn to the Intervention Group (IG) and four CMHCs to the Control Group (CG). The allocation to treatment conditions was not blinded. New referrals were screened with the AUDIT and the DUDIT. Those who scored above the cut-off level of these instruments were assessed with the Structured Clinical Interview for DSM-IV 1 and 2. We included patients with anxiety and/or depression together with one or more SUDs.

**Results:**

We included 55 patients in the IG and 21 in the CG. A linear multilevel model was used. Both groups reduced their alcohol and substance use during the trial, while there was no change in psychiatric symptoms in either group. However, the IG had a greater increase in motivation for substance use treatment after 12 months than had the CG with an estimate of 1.76, p = 0.043, CI_95%_ (0.08; 3.44) (adjusted analyses). There were no adverse events.

**Conclusions:**

Integrated treatment is effective in increasing the motivation for treatment amongst patients with anxiety and/or depression together with SUD in outpatient clinics.

**Trial registration:**

ClinicalTrials.gov: NCT00447733.

## Background

Substance use disorders (SUD) are among the most common mental disorders (MD) with lifetime prevalence between 15 and 27% [1-3]. A high comorbidity between MD and SUD is established from numerous studies [1,4-7]. This comorbidity is associated with poorer effect of treatment resulting in poorer psychosocial functioning, a higher number of days in treatment, higher attrition from treatment, more admissions, and a higher burden of disease from both their MD and SUD [8-14]. By SUD we refer to abuse and dependence of both alcohol and illegal substances.

One of the difficulties in treating this comorbidity is that treatment services may lack sufficient combined expertise to treat both types of disorders [15]. This has often resulted in sequential or parallel treatment approaches, which tend to result in poor treatment outcomes, [12,16-18]. To overcome these difficulties, the Integrated Treatment (IT) approach was developed in the United States at the end of the 1980’s [18,19] and clinical guidelines to this treatment were later described by Minkoff [20]. The main purpose of this approach is to offer the patient a combined treatment for both the MD and the SUD by the same therapist or therapeutic team, at the same site at the same time. This treatment should be comprehensive, assertive, focusing on both rehabilitation and harm-reduction, having a long-term perspective and use multiple, evidence based therapeutic modalities like Motivational Interviewing (MI) [21-23] and Cognitive Behavioural Therapy (CBT) [24,25]. Most clinical guidelines for the treatment of comorbid MD and SUD are recommending a combination of MI and CBT [26-29].

However, most of the research on the effectiveness of IT has been conducted on patients with SUD co-occurring with severe mental disorders. Less is known about the effectiveness of IT for patients with SUD co-occurring with less severe mental illnesses such as anxiety and affective disorders without psychosis. The aim of this study was to investigate the effectiveness of IT for patients with SUD together with anxiety and/or depression in psychiatric outpatient clinics on the following parameters:

1) The use of alcohol and other substances.

2) The severity of psychiatric symptoms.

3) The client’s motivation for changing his/her substance use behaviour.

## Methods

### Design

The study compares the effectiveness of Integrated Treatment (IT) to treatment as usual (TAU) in the psychiatric outpatient clinics of Community Mental Health Centres (CMHCs). To obtain external validity, we chose a pragmatic randomized controlled trial (RCT) design. In order to acquire the calculated sample-size, we chose to run a multi-centre study. As contamination of knowledge between therapists and patients between groups was an obvious risk, we decided to randomize on centre-level. Blinding was judged impossible and therefore the allocation to treatment conditions was open at inclusion. Five CMHCs were drawn to the intervention group and four CMHCs to the control group. For more details see a previous paper [30].

### Participants

Patients were sampled from psychiatric outpatient clinics at 9 CMHCs located in the south, eastern and central Norwegian Regional Health Trusts. The CMHCs are part of the specialist level treatment services located in both urban and rural parts of Norway. The patients are generally referred by general practitioners, emergency rooms and inpatient hospital departments for outpatient specialist psychiatric treatment and follow-up. People with anxiety and depression are predominantly referred by their general practitioners.

The inclusion criteria were: new referrals, above 18 years of age, anxiety disorder and/or depression with or without a personality disorder together with a disorder of abuse or dependence from drugs or alcohol. The exclusion criteria were: psychotic disorder, except episodic drug induced psychosis, planning to move away from the catchment area during the 12 months duration of the trial, not able to speak or read Norwegian, disorder of abuse/dependence of benzodiazepines or nicotine as the only substance use disorder and acute illness that required immediate treatment. Those who had an acute illness could be included in the trial after receiving the acute intervention if they were referred back to the outpatient clinic for regular treatment. Participants who provided informed consent to participate and completed the baseline assessments were included in the study.

### Sample size

At the time of planning the study there were no published effect-sizes available from studies comparing the effects of IT with TAU. We therefore computed a within-group effect-size based on changes from baseline to follow-up in the absence of a control group in a treatment study evaluating the effects of comprehensive individual and group treatment [31]. The effect-size in this study was modest (0.57), but with a 5% alpha level and 80% power, the minimum number to treat was 78 patients (i.e., N = 36 in each group). With 90% power the number was 108 patients. We expected between 20 and 30 percent dropout for this group of patients from treatment and assessments, and therefore planned to include a total of 150 patients in the study.

### Interventions

In both groups the therapists were expected to provide evidence based treatment for the psychiatric disorder of the patient, including psychopharmacological treatment. The use of such medications was therefore not a focus of the study.

The specific background and work-experience of the therapists in this study was not recorded. Generally, the therapists at CMHCs come from different backgrounds; psychologists and medical doctors in addition to nurses and social workers specialising in psychotherapy.

In the intervention group, three to five therapists at each CMHC and their local trial administrators received training in IT. This consisted of 35 hours of training in Motivational Interviewing (MI), Cognitive Behavioural Therapy (CBT), involving families and advice on pharmacological treatment. The training was repeated after 6 and 12 months. The local trial administrators and therapists were encouraged to have regular peer-group meetings at their CMHC. The investigators had regular contact with the local trial administrators by phone, e-mails and visits at the CMHCs for support.

The patients in the intervention group received IT for both their psychiatric disorder and their substance use disorder (SUD). The IT consisted of the treatment modalities CBT and MI. In addition, the therapists were to involve the patient’s family and have a more active attitude towards the patient in regard to getting the patient into treatment and continuing treatment, for example by calling or visiting the patient on “no show”. The services should also be comprehensive, i.e. that the services should be directed against a broad array of areas of functioning that are frequently impaired in clients with co-occurring mental and SUD such as housing, vocational functioning, ability to manage the psychiatric illness and family and social relationships [25]. The treatment was not manualized although a descriptive treatment guide was provided.

In the control group, the patients received treatment as usual (TAU). TAU is a difficult term to define as it depends greatly on the preference, skills, knowledge and resources of the therapists delivering it [32]. Commonly, the treatment methods used in CMHCs include psychodynamic and cognitive therapies used with an eclectic approach tailored to meet the differential needs of the individual patient. However, traditionally the treatments given at psychiatric outpatient clinics have focused mainly on the psychiatric disorders, and given little attention to the SUD.

### Procedures

We trained and paid one therapist at each CMHC to administer the project locally. These local trial administrators had 3 days of training on how to run the project and the instruments used for screening and assessments. Their scoring of case-vignettes with the EuropASI, chapter E, [33,34] was evaluated by a certified teacher. The trial administrators assessed all participants at baseline, 6 and 12 months of follow-up, while the initial screening was conducted by the therapists that the patients were referred to.

All new referrals to the psychiatric outpatient clinics of the CMHCs during a defined time period were to be screened with the Alcohol Use Disorder Identification Test (AUDIT) [35] and the Drug Use Disorder Identification Test (DUDIT) [36] to identify individuals that may have a problematic use of substances. The cut offs for the AUDIT were set to 6 for women and 8 for men [37]. The cut offs for the DUDIT were set to 2 and 6 for women and men respectively [36]. To be able to measure change in substance use during the 12 months of follow-up, the instructions for the AUDIT and the DUDIT were altered to cover substance use during the last 6 months instead of the original last 12 months. New referrals could include patients with a previous treatment history at the CMHC.

Those who scored above the threshold level on either of the screening instruments were to be referred to the local trial administrator for further assessments. Firstly, the Structured Clinical Interviews for DSM-IV axis 1 and axis 2 disorders (SCID 1 and SCID 2) [38,39] were used to assess whether the patients fulfilled the inclusion criteria. The patients that fulfilled the inclusion criteria without fulfilling any exclusion criteria were included in the study.

At baseline and both follow up interviews the included patients were assessed with the Symptom Check List 90 (SCL-90R) [40-42], the European Addiction Severity Index (EuropASI), chapter E, the Health of the Nation Outcome Scale (HoNOS) [43,44], the Alcohol Use Scale (AUS), the Drug Use Scale (DUS) [45,46], the Global Assessment of Functioning Scale (split version) (GAF) [47,48] and the Substance Abuse Treatment Scale (SATS-r) [49]. The assessments with the AUDIT and the DUDIT were repeated at 6 and 12 months of follow up. The SCID 1 and the SCID 2 were conducted at inclusion only.

For more details see a previous article [30].

### Outcome measures

To examine the change in the use of substances (alcohol and illegal drugs) during the course of the trial, we used the AUDIT and the DUDIT to assess changes during the last six months and the EuropASI to assess changes during the last 30 days. The response variables from the EuropASI were coded in the following way: ASI-Alcohol = the number of days using alcohol on a regular basis during the last 30 days (question E1 in the EuropASI manual), ASI-Illegal substances = the number of days using any illegal substances during the last 30 days (a sum of the number of days used from question E3-E12 in the EuropASI manual).

To examine the change in psychiatric symptoms in regard to anxiety and depression during the course of the trial, we used the sum scores of the SCL-90R anxiety, depression and general severity indexes.

Integrated Treatment is motivation-based, i.e. adapted to the patient’s motivation for change. This approach is based on the Stages of Change [50,51] and the closely related Stages of Treatment [52]. We therefore examined how the patient’s motivation for changing substance use behaviours changed during the trial. To measure this we used the Substance Abuse Treatment Scale (SATS-r).

The outcome measures were examined on the individual level.

### Data analyses

When comparing the background variables of the two groups the Student’s t-test was used for normally distributed continuous variables and the Mann-Whitney-U test was used for skewed continuous variables. The Pearson’s chi-square test was used for categorical variables. In cases where one or more cells had an expected count less than five, the Fisher’s Exact test was used.

To examine whether there were differences between the two groups in regard to treatment response, we used a linear multilevel model where the different response variables were modelled as a function of group and time and adjusted for covariates. The clustering in the data was accounted for by a random intercept at patient level and at centre level. The primary target of analysis was the interaction between group and time, as this indicates the different treatment responses between groups during the course of the trial. We ran both Intention to treat and Completers analyses.

The intention to treat analyses (ITT) were adjusted with *Age* and *Gender* in addition to the following variables: *Living alone, Having his/her own apartment,* and *Having compulsory school only,* as these variables showed a statistically significant difference or at least a 10 percent-point difference between groups at baseline. We continued with completers analyses in the same way and adjusted these with *Age* and *Gender* in addition to the following variables: *Being in a relationship, Having his/her own apartment, Having paid work,* and *Having compulsory school only or senior high school* as these variables showed a statistically significant difference or at least a 10 percent-point difference between the completer groups at baseline. Completers were defined as having received at least 5 sessions and having met for at least 1 follow-up interview. The reason for adjusting for variables that had a 10 percent point difference or more without showing a statistically significant difference between groups is that the material is somewhat small and we wanted to make sure to include all possible confounders.

The residuals were normally distributed for all response variables except for the DUDIT and the ASI variable for illegal substances. The analyses were performed using SPSS version 20.0 [53].

### Ethics

There was a complete discussion of the study with potential participants and written informed consent was obtained after this discussion. The study was approved by the Regional Committee for Medical and Health Research Ethics in Norway (REC-East) who approved and monitored the study, and this approval is in accordance with the Declaration of Helsinki.

## Results

### Flow of centres and participants and protocol deviations

This was a difficult study to conduct and the challenges we encountered are described in detail in a previous article [30]. Thirty-five CMHCs from 3 out of 5 Regional Health Trusts were invited to participate in the trial but only 9 CMHCs accepted. Two months into the project, one of the centres in the control group resigned. Another centre in the control group did not manage to include any patients during the time span of the trial, leaving 5 centres in the intervention group (IG) and two centres in the control group (CG).

**Figure 1:**
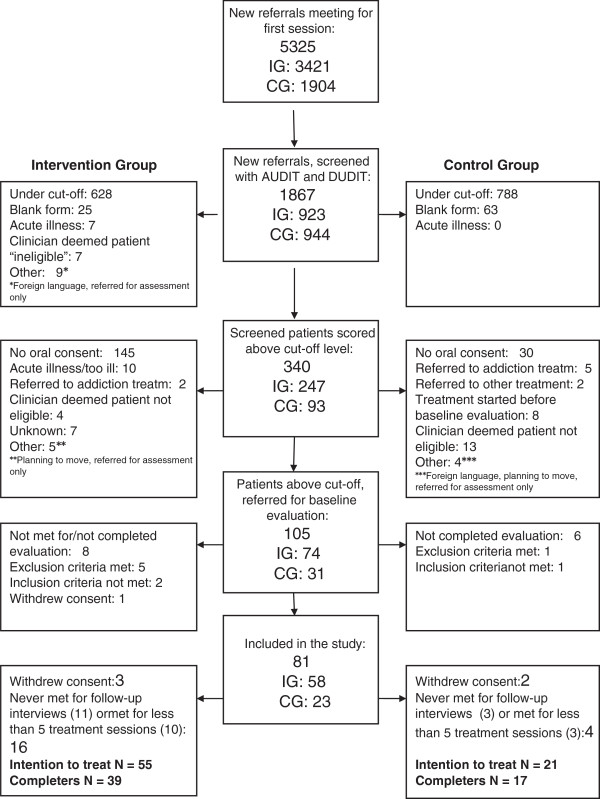
**Flowchart of participants.** Flowchart of patients in the Intervention Group (IG) and the Control Group (CG) [54].

All new referrals were to be screened with the AUDIT and the DUDIT. However, only 35% of the new referrals were screened. Eighteen per cent of the screened patients scored above the cut-off level of the screening instruments, and only 31% of these patients were referred to the local trial administrator for the baseline evaluation (Figure 1). All these challenges delayed the project and are thoroughly discussed in a previous paper [30].

The initial recruiting period was from April until December 2007 and the follow-up period continued one year after inclusion. As recruitment of patients proved to be slow, the inclusion period was extended by one year. In the end only 76 patients, 55 in the IG and 21 in the CG (ranging from 6 to 16 patients between centres) were enrolled.

After inclusion, 16 patients in the IG and 4 patients in the CG received less than 5 sessions and/or never returned for follow-up interviews. This left 56 completers (CG: 17, IG: 39) (Figure 1). There was no significant difference in the number of completers between groups ^2^ = 0.80; df = 1; p = 0.374). There was also no significant difference in the number of sessions (CG: mean 18.9, SD 13.5; IG: mean 14.5, SD 11.3, student’s T-test. -1.4; df = 74; p = 0.160) or in the distribution of sessions received between groups. In both groups the majority of patients received 10 sessions or more (IG: 35 (63.6%), CG: 16 (76.2%) (data not shown).

Comparing completers to non-completers, we found that there were no differences between completers and non-completers in regard to the amount of substances used or the severity of psychiatric symptoms at intake as measured by the outcome measures at baseline (i.e. the AUDIT, the DUDIT, the EuropASI, and the SCID-90r) (data not shown).

### Baseline demographics

Table 1 shows the baseline demographics and clinical characteristics between groups in the ITT analyses, i.e. all the included patients. There is a statistically significant difference in age between groups; otherwise, there are no differences between the groups at baseline. About half the patients are male and have paid work. The majority of patients are not in a relationship, are living with someone and have compulsory school only or senior high school. Regarding their diagnoses, the majority of patients have an alcohol use disorder and about half the patients have a personality disorder in addition to their mood and/or anxiety disorder.

**Table 1:** Baseline demographics – intention to treat analyses

	**Intervention group**^**a**^	**Control group**^**a**^	**Statistic (p-value)**^**b**^
	**N = 55**	**N = 21**	
Background variables			
Age	32.3 (1.39)	42.2 (3.08)	−2.93 (0.007)
No. of children below 18 years	0.0	0.0	z: -0.366 (0.715)
Gender (male)	28 (50.9)	12 (57.1)	0.24 (0.626)
In relationship (yes)^1^	18 (32.7)	8 (38.1)	0.20 (0.659)
Living alone (yes)	20 (37.0)	10 (47.6)	0.71 (0.401)
Nationality (Norwegian)^2^	53 (96.4)	20 (95.2)	0.05 (1.000)^c^
Having children in pat.’s care	14 (25.5)	4 (19.0)	0.35 (0.764)^c^
Has his/her own apartment^3^	44 (80.0)	19 (90.5)	1.18 (0.496)^c^
Has paid work^4^	27 (49.1)	10 (47.6)	0.01 (0.909)
Compulsory school only^5^	16 (29.1)	2 (9.5)	3.22 (0.129)^c^
Compulsory school only or senior high school^5^	46 (83.6)	16 (76.2)	0.56 (0.514)^c^
Diagnoses			
Alcohol use disorder	43 (78.2)	17 (81.0)	0.07 (0.791)
Drug use disorder	23 (41.8)	7 (33.3)	0.46 (0.499)
Mood disorder	41 (74.5)	16 (76.2)	0.02 (0,882)
Anxiety disorder	46 (83.6)	16 (76.2)	0.56 (0.514)^c^
Personality disorder	30 (54.5)	10 (47.6)	0.29 (0.589)
Other mental disorders	3 (5.5)	4 (19.0)	3.36 (0.087)^c^

The baseline demographics and clinical characteristics between groups in the Intention to Treat analyses.

^a^“Age” is presented as mean (SD) and “no. of children below 18 years” is presented as median. All other variables are presented as n (%). Valid percentages are given.

^b^The student’s T-test is used for “age” and the Mann-Whitney-U-test is used for “no. of children below 18 years”. For all other variables the χ^2^-test is used.

^c^Where one or more cells have an expected count less than five the 2 sided Fisher’s Exact test is used.

^1^“In relationship” = married or cohabitant. Not in relationship = unmarried, widow (er), separated or divorced.

^2^“Non-Norwegian” = Afghan, Swedish or German.

^3^“Not having his/her own apartment” = living with parents or friends.

^4^“No paid work” = being provided for, student loan, unemployment benefit, sickness or rehabilitation allowance, disability pension, retirement pension, National Assistance, other.

^5^“Education”: Compulsory school (primary and secondary school), Senior high school (upper secondary school), Higher education (college or university).

Looking at the baseline demographics and clinical characteristics amongst the completers, there is a statistically significant difference between groups in regard to *other mental illness* (IG: 1/39, CG: 4/17), otherwise the pattern of the baseline characteristics of the completer group is identical with that of the ITT-group (data not shown).

### Summary of the results

From the ITT analyses (Table 2) we see that there is a statistically significant reduction in the use of alcohol as measured by the AUDIT in the CG between baseline and 12 months (*12 months*). However, the additional reduction of alcohol use in the IG is not statistically significant (IG*12 m). There is also a statistically significant reduction in the use of illegal substances as measured by the DUDIT in the CG between baseline and 12 months (*12 months*). Yet, the additional reduction in the IG is not statistically significant. There is also a statistically significant reduction in the use of alcohol as measured by the ASI in the CG between baseline and 6 months (*6 months*) and between baseline and 12 months (*12 months*). However, the additional reduction in the IG during the same time periods is not statistically significant. In summary, both groups reduce their use of alcohol and illegal substances during the 12 month course of the trial, but the IG does not improve significantly more than the CG.

**Table 2:** Treatment response - intention to treat analyses

**Response variables**	**Parameter**	**Unadjusted**				**Adjusted**			
		**Estimate**	**Sig.**	**95% CI**		**Estimate**	**Sig.**	**95% CI**	
				**Lower bound**	**Upper bound**			**Lower bound**	**Upper bound**
AUDIT	6 months	−1.95	0.197	−4.94	1.03	−1.96	0.195	−4.94	1.02
	12 months	−3.11	**0.034**	−5.97	−0.25	−3.10	**0.034**	−5.96	−0.23
	IG*6 m	−2.72	0.115	−6.10	0.67	−2.63	0.139	−6.12	0.86
	IG*12 m	−2.26	0.181	−5.59	1.06	−2.18	0.209	−5.61	1.23
DUDIT	6 months	−1.36	0.459	−4.99	2.27	−1.30	0.478	−4.92	2.32
	12 months	−4.50	**0.011**	−7.97	−1.03	−4.50	**0.011**	−7.97	−1.04
	IG*6 m	−0.39	0.942	−13.61	12.83	−1.49	0.819	−17.30	14.32
	IG*12 m	0.74	0.891	−12.48	13.96	−0.29	0.964	−16.11	15.52
SCL-90-anxiety	6 months	−0.16	0.319	−0.48	0.16	−0.16	0.327	−0.48	0.16
	12 months	−0.21	0.191	−0.52	0.11	−0.21	0.194	−0.52	0.11
	IG*6 m	0.21	0.491	−0.51	0.92	0.20	0.548	−0.58	0.97
	IG*12 m	0.12	0.698	−0.60	0.83	0.11	0.744	−0.67	0.88
SCL-90-depres-sion	6 months	−0.20	0.347	−0.61	0.22	−0.19	0.374	−0.60	0.23
	12 months	−0.37	0.074	0.77	0.04	−0.36	0.081	−0.76	0.05
	IG*6 m	0.18	0.576	−0.54	0.90	0.32	0.437	−0.60	1.24
	IG*12 m	0.11	0.744	−0.61	0.82	0.25	0.546	−0.67	1.16
SCL-90-GSI	6 months	−0.19	0.185	−0.47	0.09	−0.18	0.194	−0.46	0.10
	12 months	−0.16	0.259	−0.43	0.12	−0.15	0.266	−0.43	0.12
	IG*6 m	0.26	0.350	−0.35	0.88	0.27	0.400	−0.45	0.99
	IG*12 m	0.03	0.911	−0.58	0.64	0.05	0.884	−0.67	0.76
ASI-alcohol	6 months	−5.97	**0.002**	−9.63	−2.31	−5.88	**0.002**	−9.55	−2.21
	12 months	−4.10	**0.022**	−7.62	−0.59	−3.95	**0.029**	−7.48	−0.42
	IG*6 m	−0.55	0.787	−4.53	3.44	2.47	0.210	−1.41	6.36
	IG*12 m	−2.73	0.170	−6.65	1.18	0.12	0.951	−3.67	3.90
ASI-Illegal substan-ces	6 months	−5.59	0.141	−13.07	1.88	−5.56	0.143	−13.03	1.90
	12 months	−6.39	0.091	−13.82	1.04	−6.45	0.088	−13.87	0.97
	IG*6 m	10.02	0.368	−15.36	35.39	9.98	0.364	−15.10	35.06
	IG*12 m	8.77	0.426	−16.60	34.13	8.86	0.416	−16.22	33.93
SATS-R	6 months	1.42	**0.001**	0.57	2.27	1.41	**0.001**	0.56	2.26
	12 months	2.10	**<0.001**	1.27	2.93	2.09	**<0.001**	1.25	2.92
	IG*6 m	1.59	0.070	−0.16	3.35	1.44	0.085	−0.25	3.12
	IG*12 m	1.89	**0.038**	0.14	3.65	1.76	**0.043**	0.08	3.44

Examining differences between the Intervention Group and the Control Group in regard to treatment response - Intention to treat analyses.

6 months = The change of the Control group between baseline and 6 months.

12 months = The change of the Control group between baseline and 12 months.

IG*6 m = The additional change of the Intervention group between baseline and 6 months compared to the Control Group.

IG*12 m = The additional change of the Intervention group between baseline and 12 months compared to the Control Group.

AUDIT = The Alcohol Use Disorder Identification Test, DUDIT = The Drug Use Disorder Identification Test, SCL-90-Anxiety = The Symptom Check List 90 anxiety sum score, SCL-90-Depression = The Symptom Check List 90 depression sum score, SCL-90-GSI = The Symptom Check List 90 Global Severity Index sum score, ASI-Alcohol = The European Addiction Severity Index, number of days using alcohol on a regular basis during the last 30 days, ASI-Illegal substances = The European Addiction Severity Index, number of days using any illegal substances during the last 30 days, SATS-R = The Substance Abuse Treatment Scale, revised.

Statistical significant values are written in bold types, a 5% alfa-level is used.

A linear multilevel model is used. The different response variables are modelled as a function of group and time. The clustering in the data is accounted for by a random intercept at patient level and at centre level. The primary target of analysis was the interaction between group and time. The analyses where adjusted for by the following covariates: *Age, Gender, Living alone, Having his/her own apartment* and *Having compulsory school only.* During the adjusted analyses a non-restricted Log likelihood was used. In the final models, the restricted Log Likelihood was used.

Regarding the change in psychiatric symptoms as measured by the SCL-90r, there are no statistically significant changes from baseline during the course of the trial in either group.

Regarding motivation for treatment, there is a statistically significant interaction between group and time regarding SATS-r (p = 0.003, adjusted, data not shown). Looking at Table 2, this effect is evident after 12 months in both the unadjusted and adjusted analyses (IG*12 m). This means that the IG has a greater increase in motivation for substance abuse treatment during the 12 month course of the trial than the CG.

The completer analyses show similar results as the intention to treat analyses on all parameters. Regarding motivation for treatment, there is a statistically significant interaction between group and time regarding SATS-r (p = 0.008, adjusted, data not shown). Looking at Table 3, this effect is evident after 12 months in the unadjusted analyses (IG*12 m) and after 6 and 12 months (IG*6 m, IG*12 m) in the adjusted analyses.

**Table 3:** Treatment response - completers analyses

**Response variables**	**Parameter**	**Unadjusted**				**Adjusted**			
		**Estimate**	**Sig.**	**95% CI**		**Estimate**	**Sig.**	**95% CI**	
				**Lower bound**	**Upper bound**			**Lower bound**	**Upper bound**
AUDIT	6 months	−2.20	0.141	−5.14	0.74	−2.22	0.137	−5.15	0.72
	12 months	−3.53	**0.017**	−6.41	−0.65	−3.53	**0.017**	−6.41	−0.65
	IG*6 m	−3.18	0.057	−6.46	0.099	−2.72	0.106	−6.02	0.59
	IG*12 m	−2.76	0.097	−6.03	0.51	−2.35	0.160	−5.64	0.94
DUDIT	6 months	−0.97	0.615	−4.80	2.86	−0.98	0.612	−4.81	2.84
	12 months	−4.18	**0.029**	−7.92	−0.43	−4.18	**0.029**	−7.92	−0.43
	IG*6 m	1.51	0.784	−11.80	14.82	−1.45	0.806	−15.87	12.96
	IG*12 m	2.49	0.652	−10.82	15.79	−0.48	0.935	−14.89	13.93
SCL-90-anxiety	6 months	−0.13	0.418	−0.45	0.19	−0.13	0.429	−0.45	0.19
	12 months	−0.17	0.289	−0.49	0.15	−0.17	0.285	−0.49	0.15
	IG*6 m	0.34	0.230	−0.22	0.89	0.38	0.204	−0.21	0.97
	IG*12 m	0.22	0.429	−0.33	0.78	0.27	0.372	−0.33	0.86
SCL-90-depres-sion	6 months	−0.20	0.372	−0.63	0.24	−0.19	0.374	−0.63	0.24
	12 months	−0.36	0.097	−0.80	0.07	−0.37	0.094	−0.80	0.06
	IG*6 m	0.27	0.501	−0.66	1.19	0.34	0.393	−0.57	1.25
	IG*12 m	0.18	0.646	−0.75	1.11	0.25	0.525	−0.66	1.16
SCL-90-GSI	6 months	−0.16	0.257	−0.45	0.12	−0.16	0.260	−0.45	0.12
	12 months	−0.13	0.353	−0.42	0.15	−0.14	0.345	−0.42	0.15
	IG*6 m	0.39	0.200	−0.30	1.09	0.43	0.157	−0.23	1.08
	IG*12 m	0.15	0.595	−0.54	0.85	0.18	0.511	−0.48	0.84
ASI-alcohol	6 months	−6.28	**0.001**	−9.96	−2.61	−6.40	**0.001**	−10.09	−2.72
	12 months	−4.29	**0.020**	−7.90	−0.69	−4.29	**0.020**	−7.91	−0.68
	IG*6 m	−0.72	0.723	−4.70	3.27	1.86	0.432	−3.56	7.29
	IG*12 m	−3.40	0.093	−7.38	0.58	−1.05	0.648	−6.44	4.34
ASI-Illegal substances	6 months	−5.41	0.182	−13.41	2.58	−5.41	0.182	−13.40	2.58
	12 months	−6.24	0.125	−14.23	1.76	−6.24	0.125	−14.23	1.76
	IG*6 m	10.32	0.315	−13.15	33.79	10.94	0.308	−13.90	35.78
	IG*12 m	8.90	0.381	−14.56	32.37	9.55	0.368	−15.28	34.38
SATS-R	6 months	1.65	**<0.001**	0.81	2.49	1.65	**<0.001**	0.81	2.49
	12 months	2.35	**<0.001**	1.51	3.19	2.35	**<0.001**	1.51	3.19
	IG*6 m	1.76	0.070	−0.19	3.71	1.63	**0.050**	0.001	3.25
	IG*12 m	2.18	**0.034**	0.22	4.14	2.07	**0.020**	0.44	3.70

Examining differences between the Intervention Group and the Control Group in regard to treatment response – Completers analyses.

Completers = Having received at least 5 sessions and met for at least one follow-up session.

6 months = The change of the Control group between baseline and 6 months. 12 months = The change of the Control group between baseline and 12 months. IG*6 m = The additional change of the Intervention group between baseline and 6 months compared to the Control Group. IG*12 m = The additional change of the Intervention group between baseline and 12 months compared to the Control Group. AUDIT = The Alcohol Use Disorder Identification Test, DUDIT = The Drug Use Disorder Identification Test, SCL-90-Anxiety = The Symptom Check List 90 anxiety sum score, SCL-90-Depression = The Symptom Check List 90 depression sum score, SCL-90-GSI = The Symptom Check List 90 Global Severity Index sum score, ASI-Alcohol = The European Addiction Severity Index, number of days using alcohol on a regular basis during the last 30 days, ASI-Illegal substances = The European Addiction Severity Index, number of days using any illegal substances during the last 30 days, SATS-R = The Substance Abuse Treatment Scale, revised.

Statistical significant values are written in bold types, a 5% alfa-level is used.

A linear multilevel model is used. The different response variables are modelled as a function of group and time. The clustering in the data is accounted for by a random intercept at patient level and at centre level. The primary target of analysis was the interaction between group and time. The analyses where adjusted for by the following covariates: *Age, Gender, Being in a relationship, Having his/her own apartment, Having paid work* and *Having compulsory school only or senior high school.* During the adjusted analyses a non-restricted Log likelihood was used. In the final models, the restricted Log Likelihood was used.

### Adverse events

There were no adverse events related to this project. However, one patient included in the IG who was assessed as having a severe SUD and therefore referred to a private addiction treatment centre after receiving 3 sessions at the CMHC, died about 8 months later of an overdose at the private addiction treatment centre between the 6 and 12 month follow-up interviews. He is included in the ITT analyses and regarded as missing at 12 months.

## Discussion

This study compared Integrated Treatment with TAU amongst patients in psychiatric outpatient-clinics with anxiety and/or depression together with SUD. Our main findings are that both groups reduce their use of alcohol and other substances, and that the motivation for treatment improves significantly more in the intervention group.

Our first finding is that both treatment groups show a statistically significant decline in the use of alcohol as measured by the AUDIT and the EuropASI and in the use of other substances as measured by the DUDIT. This could mean that both interventions are effective in reducing the use of these substances and that it is more important to receive treatment than which treatment is received. It may also be an effect of the assessment itself and thereby blurring experimental contrast [55,56]. On the other hand, it may also be a result of type 2 statistical error, as our sample size is quite small. A review from 2009 found a statistically significant reduction in the use of alcohol and/or other substances with the use of Integrated Treatment amongst people with SUD and co-occurring anxiety and/or depression [57] and the use of integrated depression and alcohol treatment with CBT as the main modality has shown a greater reduction in the use of alcohol compared to TAU in patients comorbid of depression and alcohol use disorder [58,59].

Our second finding is that neither group experienced a significant reduction in psychiatric symptoms. However, the follow-up time of this study was relatively short. A review from 2008 showed that there were improvements in mental health in the long term when MI was combined with CBT [26], which means that these changes may appear later in the course of treatment . It is also possible that the SCL-90r is not sensitive for small changes, especially in this small material. The use of integrated depression and alcohol treatment with CBT as the main modality has shown a greater improvement compared to TAU in depressive symptoms in patients comorbid of depression and alcohol use disorder after 3 and 6 months of follow up [60,61].

Our final finding is that the intervention group improves significantly in the motivation for treatment. This indicates that Integrated Treatment increases the patients’ motivation to change their addictive behaviours even though we fail to find an additional reduction in the use of alcohol and other substances in the IG. The reason may be that the motivation for change occurs before an actual change in behaviour which may occur later. Several studies have shown that interventions including Motivational Interviewing as one of the therapeutic components have a positive effect on the patients’ motivation for treatment and changing addictive behaviours [26,60]. Saunders found that even a brief motivational intervention of a 1 hour session was more likely to make a positive shift in the stages of change measure among opiate users at a methadone clinic than in the control group [62]. A review from 2008 showed some effectiveness in the reduction of substance use in the short term when MI was used alone, and that there were improvements in mental health in the long term when MI was combined with CBT [26].

This study has several limitations. Firstly, the sample size is quite small which indicates the possibility of a type 2 statistical error. This is a common problem in this type of treatment research as most randomized clinical trials fail to enrol the target number of patients during the target amount of time [62,63]. This problem is even more evident in research involving people with SUD that commonly present with high attrition rates from both treatment and clinical trials [64,65]. Looking at a recent review on integrated psychosocial interventions for patients presenting with co-occurring anxiety and/or depression with SUD, all the included studies had small sample sizes [57]. Secondly, as most new referrals were not screened we lost many potentially eligible participants. We have no way of comparing non-responders with responders. However, we have no indication that there was a systematic selection of those who were screened and those who were not. The representativeness of the participants from the sample of new referrals should therefore not be compromised. Thirdly, we did not focus on the use of psychopharmacological treatment during the course of the trial. If the use of such medications differed between groups, this would alter the results. However, the therapists of all CMHCs are obliged to deliver evidence based treatment for each condition including psychopharmacological treatment. Another limitation is that we did not have a good measure for treatment fidelity. This means that we do not know if the patients in the IG actually received a different treatment than those in the CG. Many studies have shown challenges in implementing new procedures [66,67]. On the other hand, there was a significant improvement in motivation for treatment in the IG which indicates that there was a difference in the interventions given between groups. Further, one could argue that the trial mainly measures the effectiveness of motivation as patients were classified as completers if they had met at as few as five sessions. On the other hand, in both groups the majority of patients received 10 sessions or more, although such complex conditions might need longer treatment and observation time than the one year follow-up of this and most other trials. Finally, as this is a group randomized trial consisting of only 7 centres, the results could be biased if one of the centres would perform much better or much worse than the other centres. To handle this potential bias, we have used a random intercept at centre level in our linear multilevel model.

To judge whether this intervention is cost-effective given the extra training needed to deliver it, one will need larger studies and a longer time of follow-up.

### Interpretation of results

As we found a decline in substance use in both groups, common therapeutic factors are demonstrated but it is unclear whether integrated treatment is more effective in reducing substance use. This might be explained by insufficient power. On the other hand, there was a significant improvement in motivation for treatment in the IG which supports that Integrated Treatment is a fruitful approach when the patient is comorbid with SUD and psychiatric disorders, not only severe mental disorders, but also milder conditions dominated by anxiety and depression. However, these findings should be seen as preliminary and confirmed in larger studies before further conclusions can be drawn.

### Generalizability

To provide external validity, we chose a pragmatic RCT design with wider inclusion criteria and few exclusion criteria. The results therefore should be generalizable to the average adult patient population in CMHCs with co-occurring anxiety and/or depression together with SUD.

This article is to a large extent structured as recommended by the Consort group for reporting on group RCTs [68-75].

## Conclusions

Integrated treatment is effective in increasing the motivation for treatment amongst patients with anxiety and/or depression together with SUD in outpatient clinics.

## Abbreviations

ASI: Addiction severity index; AUDIT: Alcohol use disorder identification test; CBT: Cognitive behavioural therapy; CG: Control group; CI: Confidence interval; CMCH: Community mental health centre; DSM-IV: Diagnostic and statistical manual of mental disorders, 4^th^ edition; DUDIT: Drug use disorder identification test; EuropASI: Addiction severity index, European version; ICD-10: International statistical classification of diseases and related health problems, 10^th^ edition; IG: Intervention group; IT: Integrated treatment; ITT: Intention to treat analyses; MI: Motivational interviewing; RCT: Randomized controlled trial; SATS-r: Substance abuse treatment scale revised; SCID-I: Structured Clinical Interview for DSM-IV, axis 1 disorders; SCID-II: Structured Clinical Interview for DSM-IV, axis 2 disorders; SCL-90r: Symptom check list 90 items revised; SPSS: Statistical package for the social sciences; SUD: Substance use disorder (including abuse and addiction from both alcohol and illegal substances); TAU: Treatment as usual

## Competing interests

The authors declare that they have no competing interests.

## Authors’ contribution

All the authors fulfil the Vancouver requirements for authorship. RG and HW have been involved in the conception and design of the study. RG and LW have been involved in the acquisition of the data. HW and LW have been involved in interpreting the data. LW has drafted the manuscript. All authors have been involved in revising the manuscript critically for important intellectual content and approved the version to be published.

## Authors’ information

LW is a psychiatrist and PhD-fellow at the Norwegian Centre for Addiction Research at the Institute of Clinical Medicine, the University of Oslo, and a medical advisor at the Agency of Welfare and Social Services, the City of Oslo. HW is a psychiatrist and professor emeritus at the Norwegian Centre for Addiction Research at the Institute of Clinical Medicine, the University of Oslo. RG is a psychologist, Head of the Department of Research and Development at the Alcohol and Drug Treatment Health Trust in Central Norway.

## Pre-publication history

The pre-publication history for this paper can be accessed here:

http://www.biomedcentral.com/1471-244X/14/67/prepub
